# Morphological and radiological mapping of dental cusps in relation to spatial constraints on tooth shape of one-humped camel (*Camelus dromedarius*)

**DOI:** 10.1186/s40851-023-00213-w

**Published:** 2023-06-19

**Authors:** Atef M. Erasha, Mohammed Nazih, Safwat Ali, Mohamed Alsafy, Samir El-gendy, Ramy K. A. Sayed

**Affiliations:** 1 Department of Anatomy and Embryology, Faculty of Veterinary Medicine, University of Sadat City, Sadat, Egypt; 2 Department of Anatomy and Embryology, Faculty of Veterinary Medicine, New Valley University, New Valley, Egypt; 3 Department of Anatomy and Embryology, Faculty of Veterinary Medicine, Minia University, Minia, Egypt; 4 Department of Anatomy and Embryology, Faculty of Veterinary Medicine, Alexandria University, Alexandria, Egypt; 5 Department of Anatomy and Embryology, Faculty of Veterinary Medicine, Sohag University, Sohag, Egypt

**Keywords:** Camel, Computed tomography, Cusps, Molar teeth, Morphology

## Abstract

A significant number of researches in veterinary study have been focused on dental structure; however, there are few on the orientation and identification of their cusps. Therefore, the present article aimed to spotlight the arrangement pattern of dental cusps in the camel as a folivorous and graminivorous animal. This study was conducted on eight heads of adult, healthy camels of both sexes, collected from slaughter houses. To determine the exact orientation of cusps of molar teeth, additional radiological and CT scans were performed on the mandible as a landmark that should facilitate the reading of the cusps map. It was evident that the cusps are arranged in crescentic appearance, selenodontal form, with two cusps on each side, paracone and hypocone on the lingual surface and protocone and metacone on the vestibular aspect. Thus, camels cannot wear bite like equines, which would interfere with their constant chewing method. The camels’ dental cusps provide some of the finest examples of convergent evolution, which offer insights both into correlates between form and function, and into the ability of euthomorphic cusps in intrapability and stabilization of food items and their comminution between formidable cusps and occlusal spillway in between. Further studies should be done on the brachydont teeth and tropospheric cusps to fill the functional anatomy gap of teeth, in addition to diversity of cusps form. This study is considered a basic comparative anatomical study for normal healthy dentition and forensic practice, in addition to its importance in detection of more local aspects of dental problems in camels.

## Background

Dental research in mammals has attracted attention of many authors among human and veterinary practice [1–3]. Like the roles of teeth in food prehension, mastication and body fighting, the food size affects the teeth morphology, which consequently reflects their function [4–7]. Since 33 million years ago, the cusps of horses’ teeth have been adeptly changed from fruit eating to sharper and pointed according to the leaves diet, as the climate was changed from rainy forests into cool ones. Accordingly, morphological changes occurred on the cusps, and the crown length was markedly increased as a response to harsher diet [8–10]. Moreover, the teeth shape and number vary according to the animal age and breed [11]. Likely, the teeth form, structure and function were varied among mammals [12]. The dental shape and structure indicate the type of food which animal are capable of eating [13–15].

The mammalian teeth are categorized as: Incisors, Canines, Premolars and Molars. Each tooth consists of the enamel coated part, the crown and cement-coated one, and the root. The neck is the junction between them as well as the dentine and pulp [1, 16]. The teeth are five sided: labial toward the lips, buccal toward the cheek, lingual for the surface facing the tongue and masticatory for the surface that contacts the tooth of the opposite jaw. The contact surface lies between the neighboring teeth on the same dental arch [1].

The crown is classified as anatomical and clinical; the former describes the part of dentine that is enamel coated, while the clinical is the exposed part of the tooth above the gum level [16]. The developmental stages of teeth among mammals erupt an elevated calcified point, the cusps [17]. The dental cusps of human crowns are cone–shaped volcanos and the crowns are bunodont. The horses have lophodont molars, where the cusps are joined to form lophs and the enamel is arranged in bands with infiltrated dentine. The cattle have selenodont teeth, which are similar to those of the horses, but the enamel bands are arranged in crescentic shape [18]. The short length crowns are brachydont and are adapted for soft diet and the hypsodont ones for long crowns are suitable for harder and dry food. Although previous radiological studies have been performed on the teeth of camel and equines [19–22], the dental cusps configurations have been neglected by many researchers [23, 24]. Surprisingly, we are not aware of any studies addressing the morphological characteristics of camel teeth, and therefore, the present work aimed to provide more anatomical knowledge about the teeth of one-humped camel, in addition to clarifying the arrangement pattern of its dental cusps.

## Materials and methods

This study was performed according to the regulations of Institutional Animal Care and Use Committee (IACUC) of the faculty of Veterinary Medicine, University of Sadat City, Egypt. The Ethical approval number of the study is VUSC-005-1-23.

### Animals

Eight heads of adult, apparently healthy of one-humped camel of both sexes (four animals for each sex), with an average age of 1–5 years old were collected from the slaughterhouse of the New Valley government. The samples are distributed into two groups; formalin fixed and dried bony ones as four heads for each.

### Gross morphology

The heads were cut at the level of the laryngeal region and were injected with 10% formalin fixative solution through the common carotid artery. The fixed heads were left for 24–48 h and were then immersed in the same fixative solution for longterm preservation.

For preparation of dried bony samples, the heads were immersed in suitable containers containing 40% KOH dissolved in water. The containers were then heated to boiling temperature for about 4–5 h. The heads were thoroughly washed with running water and dried for examination.

The lower jaw was separated from its muscular attachments for examination of the cheek teeth of both superior and inferior dental arches. The study included teeth crowns surfaces; buccal, lingual and occlusal, with special reference to the anatomical features of the dental cusps.

### Computed Tomography (CT)

Four mandibles were subjected to computed tomographic (CT) scan examinations within a period of 2 h after sample collection. The images were obtained using scanning conditions 20 mA and 120 kV, in addition to window width and level (W/L) of 1600/1000 using a Siemens 16 slice Somatom Emotion CT scanner. The transfer syntax language was used in DICOM to describe the DICOM file format, and the network transfer method was the little-endian.

### Morphometrical analysis

Morphometrical measurements of the molar teeth including length, width, and masticatory surface area were performed using by using Precision Digital Vernier Caliper. Data were expressed as mean ± *SEM*.

## Results

As observed in this study, there are no remarkable differences in size and arrangement of dental cusps between male and female animals. The extra gingival part of the teeth of one-humped camel is categorized according to their dental cusp’s features as selenodont type. The masticatory surface of the cheek teeth shows different protruded eminences, the cusps, which are characterized by their resemblance to crescentic shape. The cusps are accommodated to the nature of the animals’ food and the teeth alignment between superior and inferior dental arches. The dual action between the dental cusps, teeth relations and mechanics of mastication affect teeth–food interaction.

**Fig. 1:**
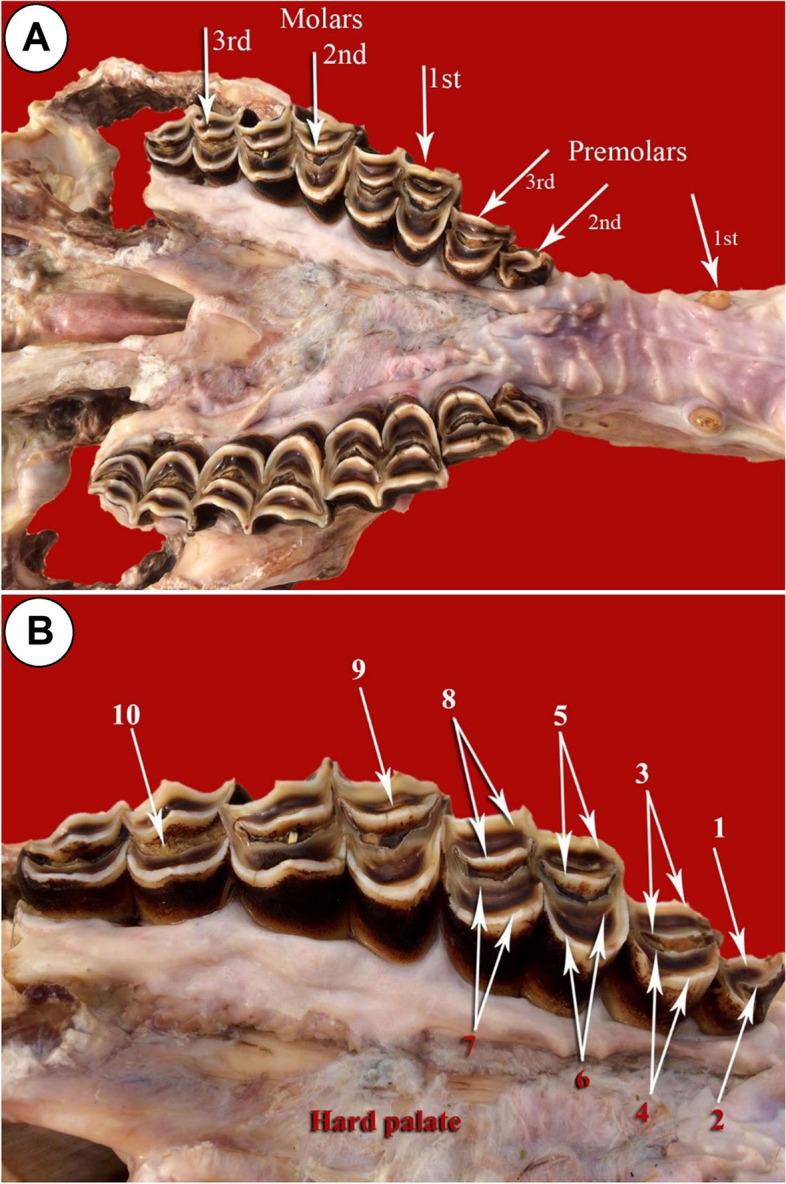
**A** Photograph showing the upper dental arch of camel (Masticatory view). **B** Photograph showing the upper cheek teeth of camel (Masticatory view). 1- Dental cusp (buccal layer); 2- dental cusp (lingual layer); 3- buccal cusp of 2^nd^ premolar; 4- lingual cusp of 2^nd^ premolar; 5- Paracone cusp of 1^st^ molar; 6- Protocone cusp of 1^st^ molar; 7- Hypocone cusp of 1^st^ molar; 8- Metacone cusp of 1^st^ molar; 9- Lingual layer (Paracone cusp) of 2^nd^ molar; 10- Buccal layer (Protocone cusp) of 3^rd^ molar

**Fig. 2:**
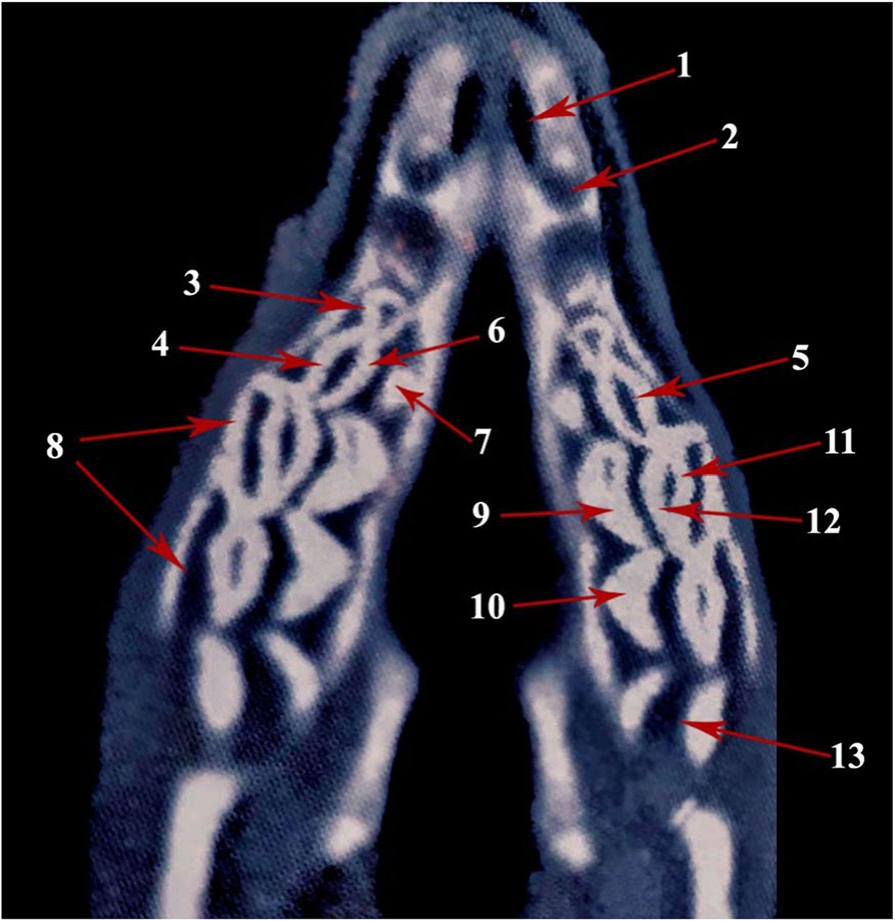
Photograph showing computed tomographic CT scan of the maxillary dental arch of camel (Masticatory view). 1- Palatine fissure; 2- Canine; 3- 2^nd^ premolar tooth; 4- 3^rd^ premolar tooth; 5- Buccal layer of the buccal cusp of 3^rd^ premolar tooth; 6- Lingual layer of the buccal cusp of 3^rd^ premolar tooth; 7- Lingual cusp of 3^rd^ premolar tooth; 8- 1^st^ molar tooth; 9- Protocone cusp of 1^st^ molar tooth; 10- Hypocone cusp of 1^st^ molar tooth; 11- Buccal layer of the paracone cusp of 1^st^ molar tooth; 12- Lingual layer of the paracone cusp of 1^st^ molar tooth

**Fig. 3:**
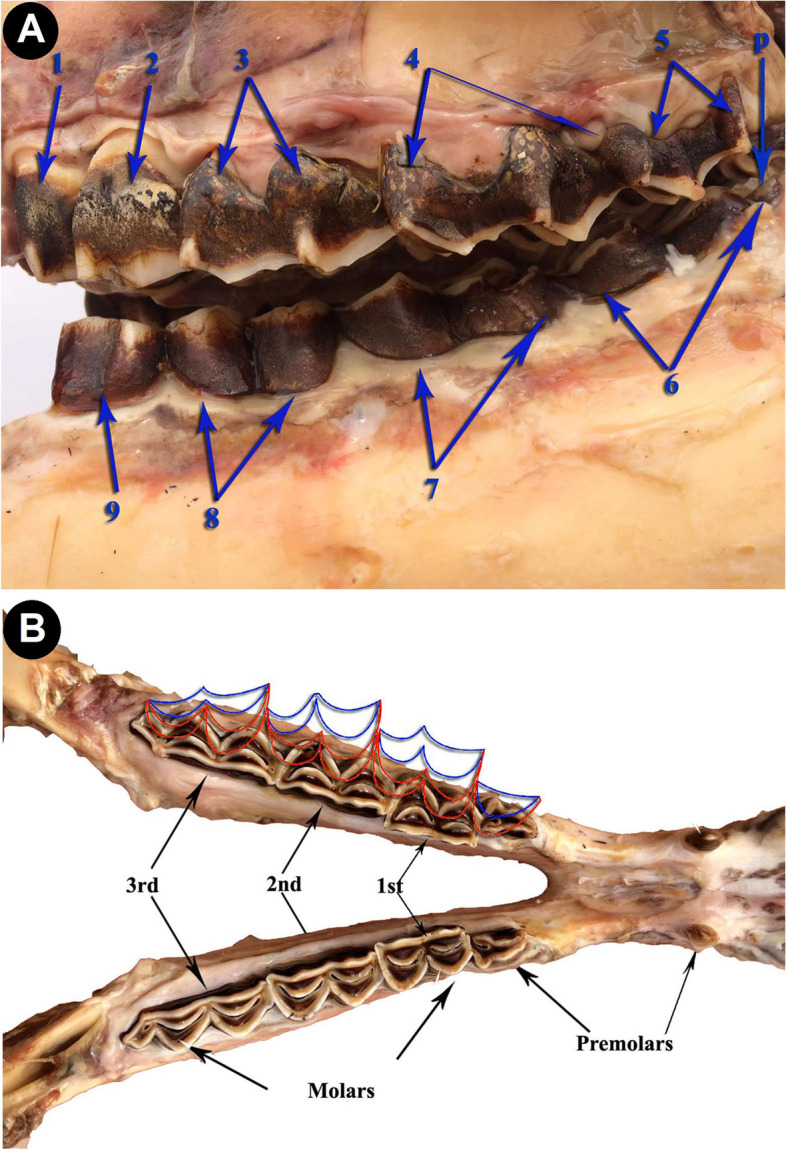
**A** Photograph showing the arrangement pattern of cheek teeth of upper and lower dental arches. (Lateral view). 1- 2^nd^ upper premolar; 2- 3^rd^ upper premolar; 3- 1^st^ upper molar; 4- 2^nd^ upper molar; 5- 3^rd^ upper molar; 6- 3^rd^ lower molar; 7- 2^nd^ lower molar; 8- 1^st^ lower molar; 9- 2^nd^ lower premolar; p- Pentaconid cusp. **B** Photograph showing the masticatory view of the mandible with an imaginary design for cusps arrangement and alignment with the maxillary ones. The blue curves indicate the outline of the upper buccal cusps border. The red curves for the lingual ones. The horizontal and vertical alignment of the dental cusps of both dental arches, fill the teeth gaps by contrary situated semicircular cutting edges. Accordingly, the cusps of cheek teeth, cover the area of mastication for massive crushing the food

**Fig. 4:**
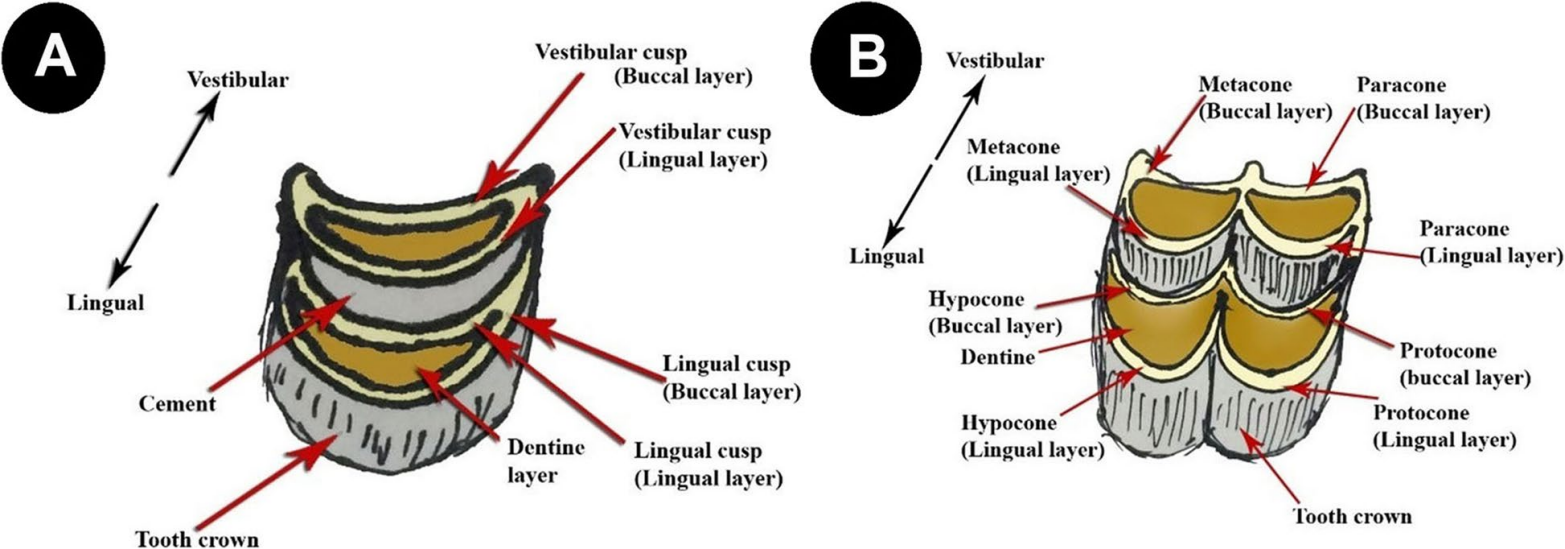
**A** Diagram showing the cusps of premolars. **B** Diagram of cusps of molars

The number of upper cheek molar teeth of camel is six in each half of the dental arch: three premolars and three molars (Fig. 1A). They are arranged in divergent attitude, where the distance between the rostral teeth increases gradually toward the caudal aspect. The premolars are smaller in size than the molars. The premolars present between the canines and the first molar tooth from mesio-distal aspect, respectively. The 1^st^ premolar is the smallest among the cheek teeth, present between the canine rostrally and the 2^nd^ premolar caudally (Fig. 1A). The tooth crown is pyramidal and directed caudo-ventrally with a single pointed cusp. The 2^nd^ premolar tooth is present on the mesial aspect of the last premolar one. The tooth crown is long and carries a single dental cusp on its occlusal surface. The last premolar is present between the 2^nd^ premolar rostrally and the 1^st^ molar caudally (Figs. 1A, B, 2 and 3A). The tooth is broader than the second premolar one, and its masticatory surface is loaded by two crescentic dental cusps, with crescentic space of cement in between (Figs. 1A, B, 2 and 4A). Each cusp is double walled: vestibular (buccal) and lingual layers and the dentine layer occupies the intervening space between the layers. The buccal cusps are slightly curved to the lingual aspect or nearly flat, while the lingual one is strongly convex toward the lingual side. The two cusps are not in the same level, where the vestibular one extends lower than the level of the lingual one (Fig. 1A, B).

The molars are larger and broader than the premolars and their corresponding sizes of the lower dental arch. Their sizes vary according to the anatomical position of each tooth (Figs. 1A, B, 2, 3A and 4B). Three sizes can be distinguished: medium, large and small in order from the mesio-distal direction. The 1^st^ molar is of medium size, and the 2^nd^ one is of large size, while the last molar tooth is the smallest (Figs. 1A, B, 2 and 3A). Their occlusal surfaces are euthemorphic with four distinct crescent cusps, which are arranged as two cusps on each tooth side, vestibular (paracone and metacone) and lingual (protocone and hypocone) (Figs. 1B, 2 and 4B). The paracone and protocone are on the mesial side and the metacone and hypocone are on the distal one. The buccal cusps extend lower than the level of the lingual ones and they are separated by two crescentic gapes of cement, mesial and distal. The cemental spaces are convex curved to the lingual side. The dental cusps are double-walled crescentic in shape, with intervening layer of dentine. Each crescent consists of vestibular (buccal) and lingual layers (Figs. 1B, 2 and 4B). They are convex toward the lingual side. The buccal layer of the vestibular cusps is nearly blunt or slightly concave. The lingual layer of the lingual cusps is more strongly convex than that of the buccal ones, and accordingly, the lingual cusps are separated by a very deep longitudinal groove. However, the buccal cusps are separated by a distinct longitudinal ridge. Generally, the lingual cusps are broader and larger than those of the vestibular side. The mesial cusps oppose the distal ones of their corresponding lower molar teeth. The paracone and protocone of the upper 1^st^ molar tooth face the metaconid and hypoconid cusps of the 1^st^ lower molar tooth, respectively. Accordingly, the metacone and the hypocone of the 1^st^ upper molar tooth face the paraconid and protoconid cusps of the 2^nd^ lower molar tooth, respectively.

**Fig. 5:**
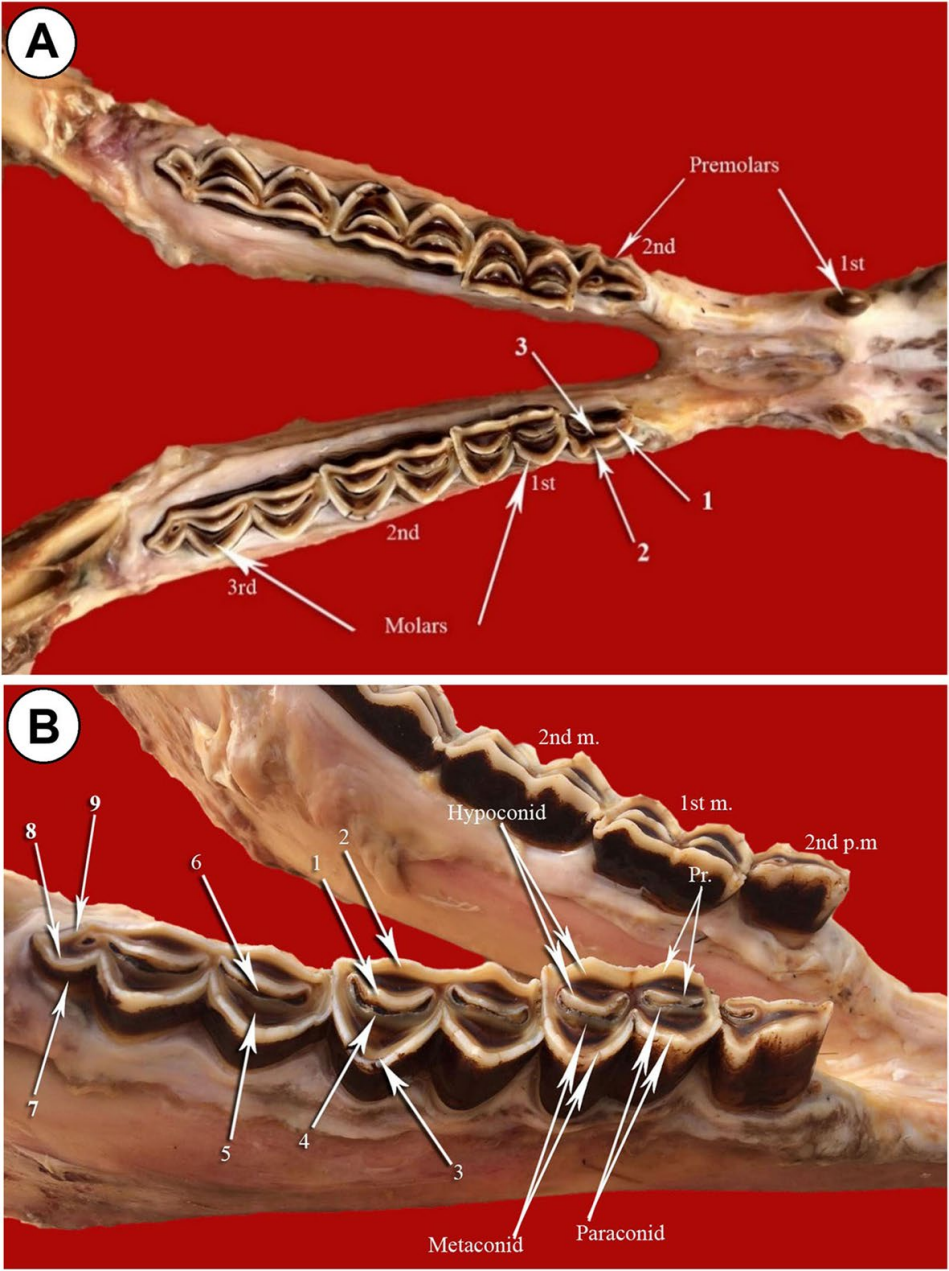
**A** Photograph showing the mandibular dental arch (Masticatory view). 1- Lingual layer of the dental cusp of the 2^nd^ premolar; 2- Buccal layer of the dental cusp of the 2^nd^ premolar; 3- layer of dentine. **B** Photograph showing the mandibular dental arch (Masticatory view). 1- Buccal layer of hypoconid (2^nd^ molar); 2- Lingual layer of hypoconid (2^nd^ molar); 3- Buccal layer of metaconid (2^nd^ molar); 4- Lingual layer of metaconid (2^nd^ molar); 5- Dentine layer of paraconid (3^rd^ molar); 6- cement layer; 7- Pentaconid; 8- Buccal layer of pentaconid; 9- Lingual layer of pentaconid; Pr- protoconid

**Fig. 6:**
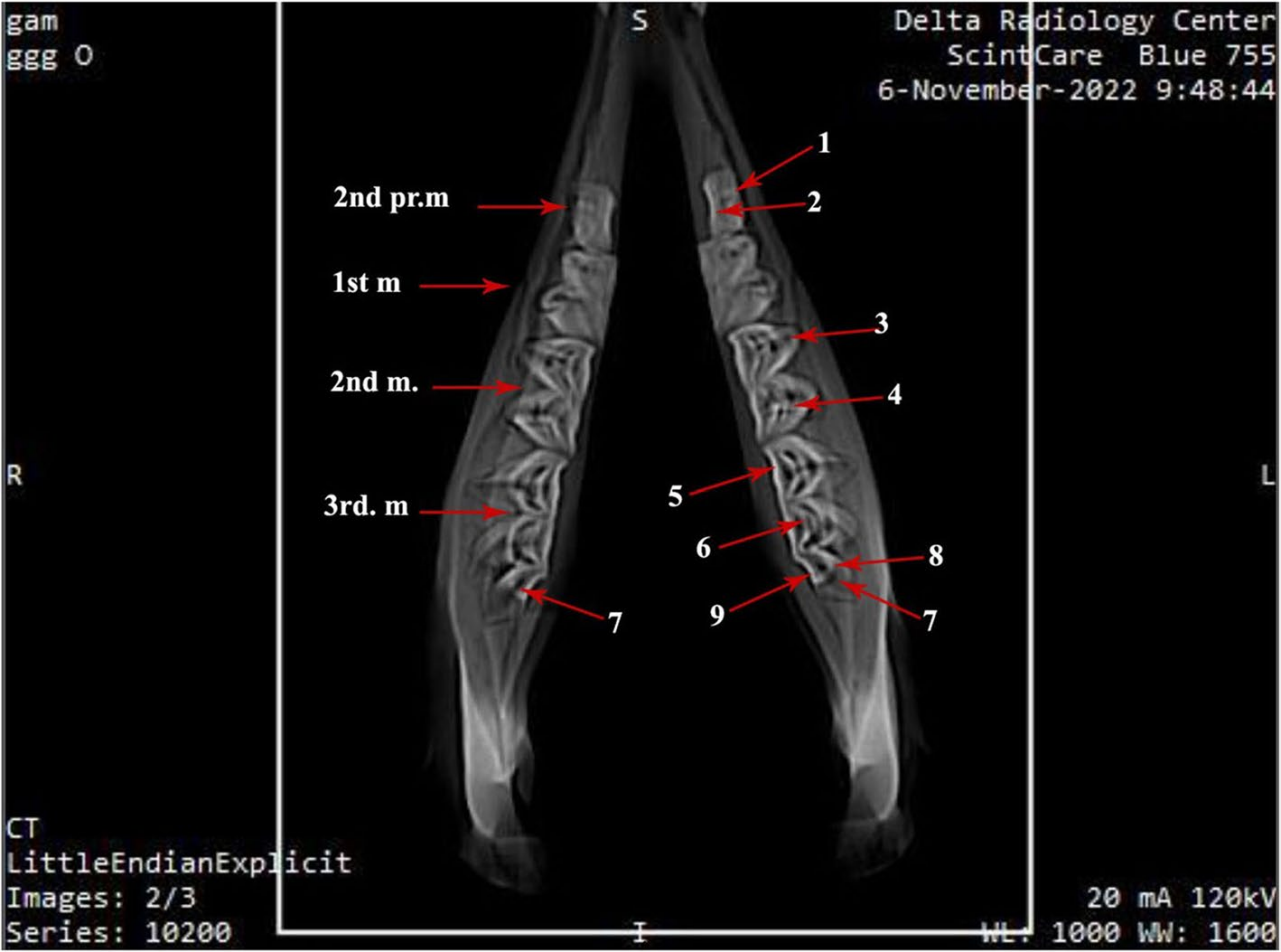
Photograph showing computed tomographic CT scan of the mandibular dental arch of camel (Masticatory view). 1- Buccal layer of the dental cusp of the 2^nd^ premolar; 2- Lingual layer of the dental cusp of the 2^nd^ premolar; 3- Buccal layer of paraconid (2^nd^ molar); 4- Buccal layer of metaconid (2^nd^ molar); 5- Lingual layer of protoconid (3^rd^ molar); 6- Buccal layer protoconid (3^rd^ molar); 7- pentaconid; 8- Buccal layer of pentaconid (3^rd^ molar); 9- Lingual layer of pentaconid (3^rd^ molar)

**Fig. 7:**
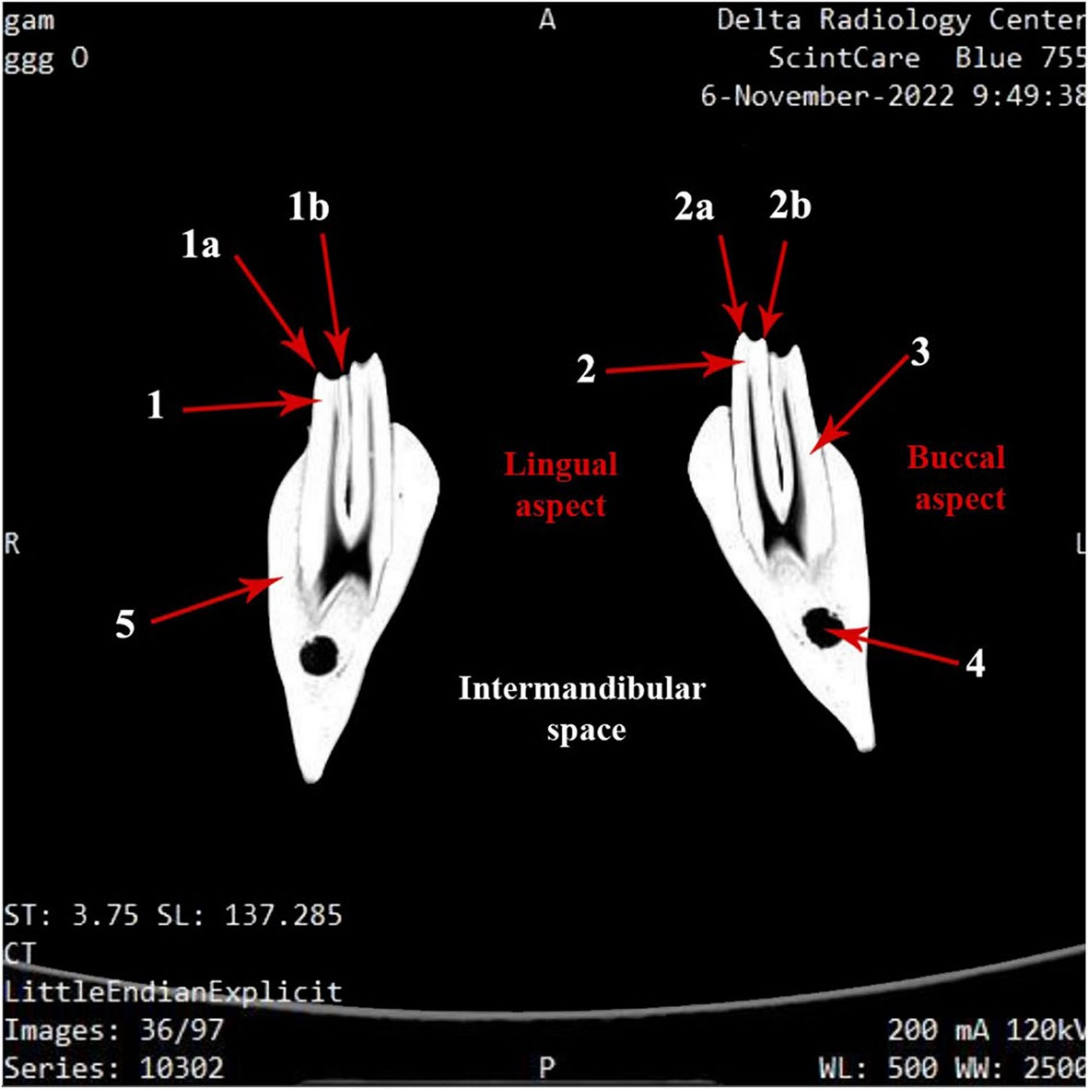
Photograph showing computed tomographic (CT) scan of the mandibular dental arch of one humped camel (Coronal section). 1- Buccal cusp; 1a- Buccal layer; 1b- Lingual layer; 2- Lingual cusp; 2a- Lingual layer; 2b- Buccal layer; 3- Mandibular tooth crown; 4- Mandibular canal; 5- Ramus of the mandible

There are two lower premolars: the 1^st^ present in the interalveolar space between the canine and the 2^nd^ one from the mesio-distal direction (Figs. 3A, 5A, B, 6 and 7). The tooth crown is short and is pyramidal in shape with caudo-dorsal curvature. It has a mesial convex border and a distal concave one. The crown bears a single pointed dental cusp. The diastema separates the tooth rostrally and the 2^nd^ premolar tooth caudally. The latter adheres to the mesial surface of the 1^st^ molar tooth (Figs. 5A, B and 6). It has compressed laterally long crown with a single crescentic shaped cusp from its occlusal aspect. The cusp is convex at the buccal surface and concave on the lingual one. It is double walled, with vestibular and lingual parts, and a layer of dentine fills the intervening space in between (Figs. 4A, 5A, B and 6).

Three massively structured molar teeth follow the last premolar one. They show gradually increasing size: small, medium and large from the mesiodistal direction (Figs. 3A, 5B and 6). The average dimensions of the first molar are 3 ± 0.2 cm in length, 2 ± 0.1 cm in width, with an average masticatory surface area of about 6 ± 0.72 cm^2^. The second molar tooth length is about 4 ± 0.1 cm and the width is 1.7 ± 0.1 cm, with the masticatory surface area of about 6.8 ± 0.5 cm^2^. The third molar tooth length, width, and masticatory surface area are 5.6 ± 0.1 cm, 2 ± 0.1 cm, and 11.5 ± 0.3 cm^2^, respectively. Their crowns are long euthemorphic with four distinct dental cusps: paraconid and metaconid on the vestibular surface and protoconid and hypoconid on the lingual one (Figs. 4B, 5A, B and 6). The last molar teeth are pentacusped with pentaconid cusp that compensates the smaller numbers of the lower cheek teeth than of the upper ones (Figs. 3A, 5A, B and 6). Generally, the cusps are double walled crescentic in structure when viewed from their occlusal surface. They have vestibular convex and lingual concave borders. The degree and size of curvature of the former cusps is higher than that of the lingual cusps. Each cusp wall has central higher and peripheral lower parts and the former is the peak of the cusp. This anatomical arrangement of cusps looks like the blade of a saw in lateral and medial views. The paraconid cusps are separated from the metaconid by a very deep vertical groove, giving the vestibular aspect its cylindrical form (Figs. 4B and 5B). The protoconid cusps are separated by a shallow groove from the hypoconid one. Double crescentic intervals of cement fill the space between adjacent vestibular and lingual cusps, the mesial and distal. The vestibular cusps have a lower level than that of the lingual side, and accordingly, the teeth masticatory surface is divided longitudinally into a higher lingual and a lower vestibular one. The upper and lower cheek teeth are different in number as the former increases by one, as will, all teeth touch together without mesial or distal gaps. The upper 2^nd^ and 3^rd^ premolar are connected to the lower 2^nd^ premolar in two sites. The upper 2^nd^ premolar is completely connected, while the mesial half of the last upper premolar touches its corresponding distal half of the lower 2^nd^ premolar. The distal half of the last upper molar is connected to the pentaconid cusp of the last lower molar (Fig. 3A, B).

**Fig. 8:**
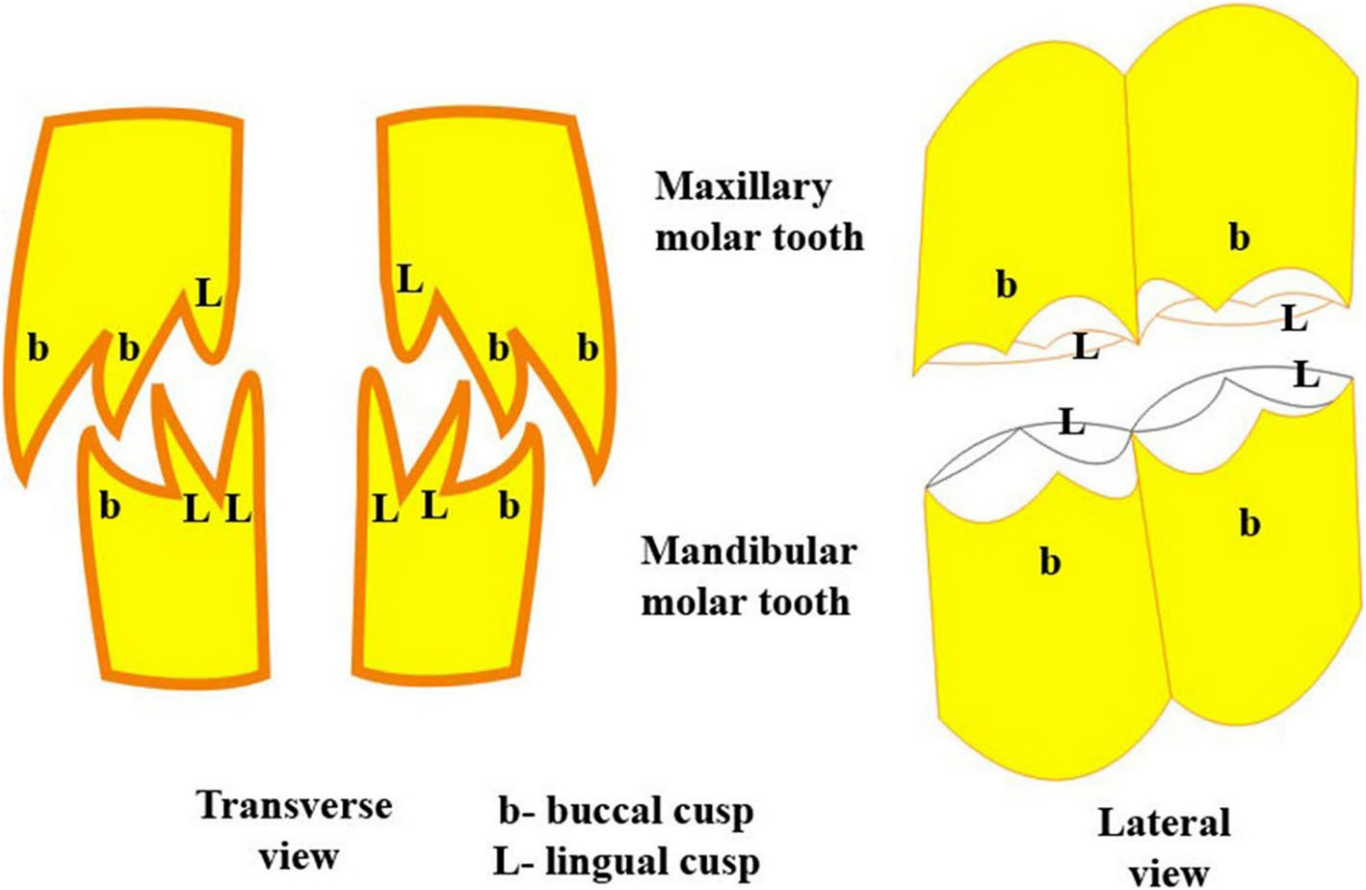
Diagram showing the arrangement pattern of the dental cusps of upper and lower cheek teeth

Generally, according to the anatomical features of the upper and lower dental arches, the upper arch is wider than the lower one and the cheek teeth of the upper arch are broader than the opposing ones of the mandibular one. Moreover, the upper cheek teeth have more numerous arches than the lower ones. These anatomical characteristics arrange the cheek teeth in both arches in an alternative manner in either the longitudinal or transverse axis. Therefore, the dental cusps of the upper cheek teeth fill the gaps between those of the lower dental arch. In the horizontal axis, the upper buccal cusps are reflected on the vestibular aspect of those of the lower arch (Fig. 8). Therefore, the camel cusps configuration that allows camel teeth to perform massive crushing action is attributed to the contrarily directed dental cusps of the upper and lower masticatory surfaces of the cheek teeth, in addition to the effect of laterally directed mastication behavior.

## Discussion

This study sheds light on the one-humped camel to fill the gap in the literature about the dental cusps configuration and its adaptation to the nature of their food. It revealed that the occlusal surfaces of the cheek teeth carry two types of cusps according to the tooth type: single pointed and modified crescentic (selenodont). The first type is present for the 1^st^ premolars of both superior and inferior dental arches, while the second type is present for the 2^nd^ and 3^rd^ premolars and molars. Regarding this aspect, cattle teeth are selenodont, with cusps resembling those of horse enamel bands, where the cusps are arranged in crescentic appearance [13]. Regarding this, recent studies showed that the crescentic cusps differed in their number and arrangement according to the tooth type [25]. The cusp consisted of a double wall of buccal and lingual enamel eminence with lunar-like appearance, where the dentine layer filled the intervening space. The described features were present singly on the occlusal aspect of the upper and lower premolars. They were double cusps: vestibular and lingual on the masticatory aspect of the upper last premolars. The cusps were separated by mesial and distal curved areas of cement, a description which has not been described in the available literature, providing a new area for understanding the morphology and function of teeth that play an essential role in sustaining life as organs of mastication [26]. Regarding another aspect, according to the herbivorous teeth configuration, horse teeth are lophodont, where the cusps are arranged in lophs, which are enamel bands with soft dentine. Furthermore, the lophs run anterio-posteriorly between the lingual and labial aspects of the tooth [27–29].

It is interesting that, in order to differentiate between the superior and inferior cheek teeth of camels, three basic items should be attended to: tooth dimensions, cusp numbers and cusps convexity direction. Concerning the former, the cheek teeth of the upper dental arch were relatively broader than those of the lower. The only teeth with double cusps were the upper last premolars. Increases in cusp number have been reported to be driven by the demands of food manipulation [30].

The anatomic-dynamic side of our study considered the dental cusps configuration together with their teeth alignment in the arch quadrant, or with their opposing ones of the opposite arch and the mechanics of mastication behavior for the one-humped camel. This was one point which was lacking among the available articles where the authors focused their work on describing the teeth cusps and their evolution [6, 31–33]. Wright et al. [33] categorized the relationship between the masticatory surfaces and chewing motions into: shearing, where crests of opposing arches slide past one another in vertical movement. The second type was crushing, where the dental cusps pressed into basins in vertical motion, while the third type was grinding, where the cusps slid on a basin in horizontal movement. An article by Ungar [7] gave an overview of the studies on mammalian tooth function and wear similar to our work. In regards to camel teeth–food interaction, the present article explained the combined interaction between undulant alignment of the cheek teeth, including their arrangement within the dental arch to their opposing ones of the opposite arch and dental arch width differences, as the upper was wider than the lower. Furthermore, camel has a broader masticatory surface of teeth with contrarily directed cusps among the dental aches. Altogether, with the lateral behavioral mastication, the teeth of camel are designed appropriately for a massive grinding effect on masticated food. These findings are parallel to those previously reported [34, 35] in studies that showed the role of herbivorous broad teeth with complex biting surfaces on the vegetation.

## Conclusions

It is obvious that the cusps of the one–humped camel are arranged in crescentic appearance, where the cusps are separated by mesial and distal curved areas of cement, which provide the morphological and functional characteristics of the camel teeth. The inability of the camel to wear bite is attributed to the presence of two cusps on each side of its teeth. Furthermore, the massive crushing action of the camel cusps is attributed to the contrarily directed dental cusps of the upper and lower masticatory surfaces of the cheek teeth, in addition to the effect of laterally directed mastication behavior. Finally, studying the morphology and structure of dental cusps of the camel is essential for understanding its behavior, handling, manipulation and safety considerations. Moreover, this study has clinical significance as it is considered a basic comparative anatomical study for normal healthy dentition and forensic practice, in addition to its importance for detection of more local aspects of dental problems in camels.

## Data Availability

Data of the current study are available from the corresponding author upon request.
