# Correction: Morphological and radiological mapping of dental cusps in relation to spatial constraints on tooth shape of one humped camel (Camelus dromedarius)

**DOI:** 10.1186/s40851-023-00216-7

**Published:** 2023-09-01

**Authors:** Atef M. Erasha, Mohammed Nazih, Safwat Ali, Mohamed Alsafy, Samir El-gendy, Ramy K. A. Sayed

**Affiliations:** 1 Department of Anatomy and Embryology, Faculty of Veterinary Medicine, University of Sadat City, Sadat, Egypt; 2 Department of Anatomy and Embryology, Faculty of Veterinary Medicine, New Valley University, New Valley, Egypt; 3 Department of Anatomy and Embryology, Faculty of Veterinary Medicine, Minia University, Minia, Egypt; 4 Department of Anatomy and Embryology, Faculty of Veterinary Medicine, Alexandria University, Alexandria, Egypt; 5 Department of Anatomy and Embryology, Faculty of Veterinary Medicine, Sohag University, Sohag, 82524 Egypt

**Zoological Letters (2023) 9:14**

10.1186/s40851-023-00213-w

Following publication of the original article [1], it came to the journal’s attention that the article had been processed with an out-of-date manuscript file, which was out of date because the quality of the article’s English still required some improvement. The published article has since been updated with the correct manuscript file, that is, the version of the file in which all necessary improvements to the quality of the English were made. In this regard, the journal and the authors would like to highlight that the scientific argument (e.g., the results and conclusion) of the article is not affected: this update is just to the quality of the English in the article.

The publisher thanks you for reading this erratum and apologizes for any inconvenience caused.

